# The salinity challenge

**DOI:** 10.1111/nph.16357

**Published:** 2020-01-02

**Authors:** Dale Sanders

**Affiliations:** ^1^ The John Innes Centre Norwich Research Park Norwich NR4 7UH UK

**Keywords:** GABA shunt, potassium nutrition, salinity energetics, salinity tolerance, sodium exclusion

## Abstract

This article is a Commentary on https://doi.org/10.1111/nph.15955; https://doi.org/10.1111/nph.15713; https://doi.org/10.1111/nph.15773; https://doi.org/10.1111/nph.15864; https://doi.org/10.1111/nph.15862; https://doi.org/10.1111/nph.15852; https://doi.org/10.1111/nph.15758.

Terrestrial plants face formidable challenges when growing in saline conditions. High osmotic pressure around the roots can prevent efficient water absorption, yet the high concentrations of the sodium ion (Na^+^) and the chloride ion (Cl^−^) must be excluded from the cytosol, where they are toxic. The issue of how plants grow on saline soils is an interesting one in its own right, but commands added urgency in the context that irrigation and sea level rises are increasing the proportion of arable land that is salinized and that global demand for more food is leading to cultivation of marginal land.

A collection of research papers and review articles in this issue of *New Phytologist* reminds us that these challenges are experienced by plants at cellular, tissue and whole organism levels. Major changes in energy metabolism are experienced by plants growing in saline conditions. Many of these changes relate to the decreased resource devoted to growth and the necessity for enhanced transport of ions that can be used to retain turgor, or to exclude those ions that leak into cells and tissues.

‘Major changes in energy metabolism are experienced by plants growing in saline conditions.’

The Tansley review by Munns *et al.* (2020a; pp. 1072–1090) provides an insightful overview of the issues relating to energy cost and salinity tolerance in crops. The authors review aspects of free energy supply (principally through that of respiration) for continued growth and transport of water and ions through the plant; the energy cost of Na, Cl and water transport through plants in saline conditions; and the impacts of root system architecture on the ability of crops to withstand salinity. The review is careful to explain not only what we know, but also to expose what we do not know, and is nicely complemented in each of the three areas it explores by more detailed papers.

Flexibility in plant metabolism can provide adaptation to saline environments. In a thoughtful analysis, Fricke (2020; pp. 1152–1165) builds on published and unpublished data from barley to conclude that respiration at night might provide a key to water‐use efficiency, and to the maintenance of growth. Understanding how this diurnal control of respiration is achieved even in nonsaline conditions is a biochemical challenge, and this article brings an interesting added dimension to the issue. Metabolic flexibility is also required in saline conditions because a number of key respiratory enzymes, including the mitochondrial 2‐oxoglutarate dehydrogenase complex, are Na‐sensitive. Che‐Othman *et al.* (2020; pp. 1166–1180) discovered that in wheat leaves experiencing salinity stress, activation of GABA metabolism can provide a by‐pass to salinity‐sensitive routes to respiratory activity – topping up the tricarboxylic acid (TCA) cycle through the production of succinate.

Plant salinity tolerance involves complex ‘decision‐making’ by a plant: for example, how is turgor maintained in an energetically‐effective way that does not compromise metabolic activity? Munns *et al.* (2020b; pp. 1091–1096) address this question in a sophisticated analysis that develops a whole‐plant perspective in relation to the costs of ionic exclusion. The interesting conclusion is that plants – crop plants at least – must exclude all but about 2% of the sodium chloride (NaCl) that surrounds them in saline conditions. Most of the NaCl that is accumulated for osmotic purposes resides in the vacuole, which is relatively metabolically inert. A question remains around the production cost of so‐called compatible solutes that are required in the cytosol for osmotic balance of high concentrations of vacuolar inorganic ions.

The process of cation absorption and compartmentation – intracellularly and through the plant – comprises a further challenge to plants in saline conditions. Rubio *et al.* (2020; pp. 1097–1104) address this issue in the context of potassium (K) homeostasis. The necessity for a high K : Na ratio in the cytosol is particularly demanding in the context of cation absorption if the transporters involved in K uptake are not particularly selective for K over Na. There is clearly an important role here for the high affinity, highly selective HKT family of K transporters in facilitating uptake, as well as the vacuole in providing a ready source of K to buffer the cytosolic concentration.

Coping with salinity stress involves not just selective exclusion and competition between Na and K: glycophytic plants possess inherent leaks in their membranes that facilitate futile cycles in transport, particularly in the case of Na. Shabala *et al.* (2020; pp. 1105–1110) consider this phenomenon in the context of vacuolar accumulation of Na: multiple Na leak pathways (channels) recycle the ion into the cytosol. The authors point to our general lack of understanding of the roles of these channels and the physiological controls that are exerted on them.

As multicellular organisms, the manner in which plants handle salinity stress cannot be considered at the cellular level alone. Arsova *et al.* (2020; pp. 1111–1119) point to the wider physiological aspects of salinity tolerance, and in particular the need to capitalize on new phenotyping methods to analyse spatio‐temporal dynamics of root development in relation to root architecture.

The articles briefly summarized here are invaluable in pointing to recent advances and discoveries in plant salinity tolerance. But more important, perhaps, are the questions relating to the big unknowns that the authors raise. There were hopes two decades ago that developing an understanding of signal transduction pathways would lead to rapid developments in generating salt‐tolerant plants (Sanders, 2000). These hopes were based on elegant genetic analyses in Arabidopsis (Wu *et al.*, 1996) that identified genes central to activation of membrane transporters and stress responses elicited by salt stress (Liu & Zhu, 1998; Liu *et al.*, 2000). Subsequent genetic analysis by Munns *et al.* (2012) based on natural diversity identified a transporter (HKT1) that is pivotal in delivering Na to the xylem of wheat and for which variation can improve grain yield in saline conditions. Exploiting natural variation should provide further clues through genome‐wide association studies (GWAS) to key variables involved in crop tolerance to salinity.

This collection of articles reminds us that salinity tolerance is multigenic and needs to be viewed in a wider context than just that of membrane transport of NaCl. Munns *et al.* (2020a) have provided a convincing framework for establishing the energetic cost of living in a saline environment. Future research directions are proposed, and one of those is computational modelling based on critical analysis advanced by the authors. This will undoubtedly be a critical component of future research, but cannot be viewed as delivering a holistic solution without further methodological approaches such as GWAS. The power of 21^st^ century genomics is ever‐growing and this – alongside the machine‐learning approaches to understanding phenotypic diversity in an environmental context – will be key to developing the predictive breeding approaches that are needed for the realization of salinity‐tolerant crops.

‘… salinity tolerance is multigenic and needs to be viewed in a wider context than just that of membrane transport of NaCl.’

Wider aspects of biodiversity should not be forgotten, either, as a way of devising strategies for crop improvement since there are many angiosperm species that are salt‐tolerant. There are clearly lessons to be learned from the physiological adaptations that enabled such species to live in saline soils and even in some cases to re‐colonize the sea.
